# Targeted Knockdown of RNA-Binding Protein TIAR for Promoting Self-Renewal and Attenuating Differentiation of Mouse Embryonic Stem Cells

**DOI:** 10.1155/2015/657325

**Published:** 2015-03-31

**Authors:** Zhe Geng, Ping Li, Li Tan, Houyan Song

**Affiliations:** ^1^Key Laboratory of Molecular Medicine, Ministry of Education, Fudan University, Shanghai 200032, China; ^2^Institute of Biochemistry and Molecular Biology, School of Basic Medical Sciences, Lanzhou University, Gansu 730000, China

## Abstract

RNA-binding protein TIAR has been suggested to mediate the translational silencing of ARE-containing mRNAs. To analyze the functions of TIAR, we established RNAi and genetic rescue assays. We evaluated the expression of neuroectoderm markers Pax6 and nestin, mesoderm markers brachyury and Flk1, and hypoblast and definitive endoderm markers Sox17 and Gata6 during EB differentiation and found that knockdown TIAR expression restrained the differentiation of E14 cells. We assessed gene expression levels of Flk-1 and VE-cadherin and observed attenuated differentiation of E14 cells into endothelial cells upon downregulation of TIAR gene expression. As such, we hypothesized an essential role of TIAR related to EB differentiation. As TIAR inhibits the translation of c-myc, we proposed that downregulation of TIAR results in restrained differentiation of E14 cells, due in part to the function of c-myc. We found that TIAR inhibited c-myc expression at the translational level in E14 cells; accordingly, a reduction of TIAR expression promoted self-renewal of pluripotent cells and attenuated differentiation. Additionally, we established that TIAR inhibited TIA-1 expression at the translational level in E14 cells. Taken together, we have contributed to the understanding of the regulatory relationships between TIAR and both c-myc and TIA-1.

## 1. Introduction

Embryonic stem cells (ESCs) are pluripotent cells derived from the inner cell mass (ICM) of embryos, before implantation [1]. They are able to maintain their “stemness” via self-renewal and differentiate* in vitro* into cell types of all three embryonic germ layers [2]. Activation of signal transducer and activator of transcription 3 (STAT3), mediated via LIF/gp130 receptors, plays an important role in maintaining mouse ESC self-renewal capabilities [3]. Moreover, in mouse ESCs, the transcription factor c-myc was described to function as a key target of LIF/STAT3 signaling and sustained myc activity maintains self-renewal in the absence of LIF [4]. However, in mouse ESCs LIF does not only activate the STAT3-mediated cascade but also induce the PI3 K-pathway and the ERK-pathway [5, 6].

TIAR (TIA-1 related protein) [7] is a part of a family of RNA-binding proteins (RBPs) [8] that plays a critical role in transcriptional and posttranscriptional regulation of gene expression. In mammalian cells, mRNAs that contain adenine/uridine regions (collectively named AU-rich elements or AREs) in 5′ or 3′ untranslated regions (UTRs) are recognized and combined with three RNA recognized motifs (RRMs) within RBPs [9, 10], resulting in transcriptional and posttranscriptional regulation.

TIAR is known to have a distinct role in mediating the translational silencing of mRNAs to which AREs are bound [11]. Whereas these mechanisms of TIAR action can lead to a general suppression of translation in the cell, there seems to be a bias towards specific subsets of bound mRNAs. For example, mechanisms of TIAR-mediated repression of translation have been investigated most extensively in cells responding to environmental stress agents. Via phosphorylation of eIF2*α* [12], TIAR can contribute to the generation of nonfunctional translational preinitiation complexes [13]. However, the function of TIAR with respect to self-renewal and differentiation of ESCs has yet to be elucidated.

Previous work [14] has demonstrated that both RNA-binding protein AU-binding factor 1 (AUF1) [15] and TIAR affect c-myc translation via ARE in human chronic myelogenous leukemia cell line K562 cells. Additionally, AUF1 and TIAR are known to control proliferation via a c-myc-dependent pathway. It is crucial then to elucidate the function of TIAR in ES cells by illustrating the regulatory mechanisms that underlie the interaction of TIAR and ARE-c-myc mRNA. Control of c-myc is essential for proper cellular function as its deregulation is central to the formation of many tumor types [16]. Accordingly, c-myc promotes self-renewal in mouse ESCs; it is hypothesized that TIAR influences self-renewal through interactions with c-myc. Whereas it is known that TIAR plays a critical role in the development of primordial germ cells (PGCs) [17], the precise relationship of TIAR and ESC self-renewal and differentiation remains unclear. Here, we seek to elucidate the function of TIAR in mouse E14 ESCs, particularly with respect to self-renewal and differentiation. We illustrate the mechanism by which TIAR regulates gene expression in E14 cells. Moreover, TIA-1 [18] has also been described as an RNA-binding protein that functions as a translational repressor. Interestingly, TIA-1 shares an 80% amino acid homology with TIAR and is downregulated by TIAR at the translational level in human cervical carcinoma (HeLa) cells [19]. An additional aim of this work is to elucidate the regulatory connection between RNA-binding proteins TIAR and TIA-1.

We used RNAi and subsequent genetic rescue to analyze the function of RNA-binding protein TIAR in mouse E14 ESCs. We demonstrated inhibition of c-myc by TIAR at the translational level in E14 cells; subsequent knockdown of TIAR in E14 cells promoted self-renewal and extended pluripotency, thus attenuating differentiation. Moreover, we confirmed that TIAR also inhibited TIA-1 expression in E14 cells. Thus, we have revealed a critical part of the regulatory relationship between TIAR and TIA-1 at the posttranscriptional level.

## 2. Materials and Methods

### 2.1. Cell Culture and* In Vitro* Differentiation

E14 ESCs were a gift from Professor Austin Smith (Wellcome Trust Centre for Stem Cell Research, University of Cambridge, Cambridge, UK). E14 cells and TIAR RNAi E14 cells were maintained on gelatin-coated dishes in the absence of feeder cells in GMEM (Sigma, St. Louis, MO), supplemented with 2 mM glutamine (Gibco, Grand Island, NY), 0.001% *β*-mercaptoethanol (Gibco), 1× nonessential amino acids (Gibco), 1% sodium pyruvate (Gibco), 10% fetal bovine serum (FBS) (Gibco), and 2,000 units/mL human recombinant LIF (R & D Systems, Minneapolis, MN).

For differentiation of E14 cells into EBs, TIAR RNAi E14 cells (4 × 10^5^ cells per mL) were plated in uncoated Petri dishes in medium containing GMEM/10% FBS without LIF. Cells grew in suspension and formed EBs. The expression of markers specific to the three germ layers was assayed via RT-PCR or qRT-PCR.

For the first stage of monolayer differentiation, E14 cells and TIAR RNAi E14 cells (1 × 10^4^ cells per mL) were cultured on collagen type-IV in GMDM medium, 10% FBS, without LIF for 4 days. In the second stage, cells were cultured on collagen type-IV in GMDM medium, 10% FBS, without LIF and containing vascular endothelial growth factor (VEGF) (PeproTech) for another 4 days.

### 2.2. RNA Interference

RNAi of TIAR was accomplished using the pSIREN-RetroQ (Clontech) RNAi System. Transfections were carried out using Lipofectamine 2000 (Invitrogen) and pSIREN-RetroQ-TIAR-shRNA plasmid, which contains a short hairpin RNA (shRNA) sequence targeting mouse TIAR mRNA. Sequences of siRNA used in silencing experiments were as follows: 5′-CCGATAGCAGAAGGGTCAA-3′ (TIAR siRNA, start position at 267, targeting the TIAR coding region CR). In consideration of the fact that TIA-1 shares an 80% amino acid homology with TIAR, the sequence alignment of the cDNA of TIAR and TIA-1 was used and sequence homology of TIAR and TIA-1 was avoided. Thus siRNA sequences selected at this position were completely targeting TIAR. Nevertheless, a siRNA containing a random nucleotide sequence (5′-GTACCTCTAGCGATCAAACGA-3′) was used as a negative control. After 48 h of incubation, cells were treated with puromycin (2 *μ*g/mL; Sigma). Single clones of puromycin-resistant cells were selected and knockdown efficiency of TIAR expression was assayed via Western blot.

For rescue assays, TIAR RNAi E14 cells were transfected with pCDNA3.1-TIAR (Hygro) vector, containing the full-coding sequence of TIAR constructs, in which a short stretch of sequence complementary to the siRNA oligonucleotide was mutated to prevent destruction of exogenous mRNA.

The target sequence 5′-CCGATAGCAGAAGGGTCAA-3′, corresponding to nucleotide positions 267–285 of TIAR, was mutated to 5′-CAGACTCCAGGAGAGTAAA-3′, leaving the amino acid sequence unchanged (GAT (aspartic acid) at codon 46 mutated to GAC; AGC (serine acid) at codon 47 mutated to TCC; AGA (arginine acid) at codon 48 mutated to AGG; AGG (arginine acid) at codon 49 mutated to AGA; GTC (valine acid) at codon 50 mutated to GTA). Transfections of pCDNA3.1-TIAR (Hygro) which contain the RNAi-resistant TIAR transgene were performed using Superfect transfection reagent (Qiagen, Valencia, CA). After 48 h of incubation, cells were treated with hygromycin (75 *μ*g/mL; Sigma). Single clones of Hygro-resistant cells were selected and the expression efficiency of TIAR was assayed via Western blot.

### 2.3. Reverse-Transcription Polymerase Chain Reaction (RT-PCR)

RNA was isolated using TRIzol (Invitrogen, Carlsbad, CA), DNase I-digested (Invitrogen), and column-purified (RNeasy MinElute Cleanup; Qiagen, Valencia, CA). First-strand cDNA was synthesized using M-MLV Reverse Transcriptase (Invitrogen). To analyze expression levels of mRNA, the amount of cDNA was normalized to housekeeping gene GAPDH. To provide negative controls and exclude contamination by genomic DNA, the reverse transcriptase was omitted in the cDNA synthesis step, and the samples were subjected to PCR in the same manner with primer sets for GAPDH and were indicated at the bottom of each figure as RT (−). PCR amplification of different genes was performed using EX Taq polymerase (Takara, Japan), with a program of 94°C for 5 min, 35 cycles of 94°C for 30 s, 54–62°C for 30 s, 72°C for 1 min, and extension at 72°C for 10 min. The amplified PCR products were analyzed via electrophoresis on a 1% agarose gel and were stained with ethidium bromide for visualization. All the procedures were performed according to the manufacturers' instructions. Primer sequences are provided in Table 3.

### 2.4. Real-Time PCR Assays

Quantitative RT-PCR was performed with Power SYBR Green PCR Master Mix (Applied Biosystems, Foster City, CA) according to manufacturer's instructions. Signals were detected with an ABI7300 Real-Time PCR System (Applied Biosystems). The relative expression level was determined by the 2-delta Ct method and normalized against ribosomal protein L19 (RPL19). Primer sequences can be found in Table 4.

### 2.5. Western Blot Analysis

After treatment, cells were harvested at the indicated times in lysis buffer (20 mmol/liter Tris (pH 7.5), 150 mmol/liter NaCl, 2 mmol/liter EDTA, 10% glycerol, 1% Triton X-100, 1 mmol/liter phenylmethylsulfonyl fluoride, and protease inhibitor cocktail (Sigma)). Protein concentrations were determined via BCA (Pierce, Rockford, IL), and proteins were resolved on 12% sodium dodecyl sulfate- (SDS-) polyacrylamide gels followed by electrophoretic transfer onto nitrocellulose membranes. The membranes were blocked with 5% nonfat dry milk in Tris-buffered saline, 0.1% Tween 20, for 2 h and incubated at 4°C overnight with primary antibodies against TIAR (C-18) (1 : 500), Flk-1 (1 : 1000), Sox17 (1 : 500), VE-cadherin (1 : 1000), and c-myc (1 : 1000) (Santa Cruz Biotechnology, Santa Cruz, CA), nestin (1 : 500, Chemicon, Temecula, CA), and TIA-1 (1 : 500, Proteintech Group). Following incubation with appropriate horseradish peroxidase-conjugated secondary antibodies, signals were detected with an enhanced chemiluminescence system (Amersham Biosciences, Piscataway, NJ).

### 2.6. Immunohistochemistry

Cells were fixed in 4% paraformaldehyde and incubated for 1 h in blocking buffer (PBS, 10% FBS, and 0.1% Triton X-100). Primary antibody Oct4 (Santa Cruz Biotechnology, Santa Cruz, CA) was diluted to 1 : 500 in the blocking buffer and was applied overnight at 4°C. After three washes in PBS, secondary antibodies that had conjugated to FITC (fluorescein isothiocyanate) (Vector, Burlingame, CA) were diluted to 1 : 200 in blocking buffer and were applied for 1 h at room temperature. The cells were washed at least three times in PBS and visualized on an Olympus inverted fluorescence microscope. For nuclear counter staining, nuclei were stained with DAPI reagent (Sigma).

### 2.7. Statistical Analyses

Statistics were analyzed using SPSS version 16.0 software. One-way ANOVA was used to compare the means of several different groups. All data were expressed as the mean ± standard error of mean (SEM). Differences with *P* values <0.05 were considered statistically significant.

## 3. Results

### 3.1. TIAR Knockdown E14 Cells Maintained Self-Renewal and Pluripotency

**Figure 1:**
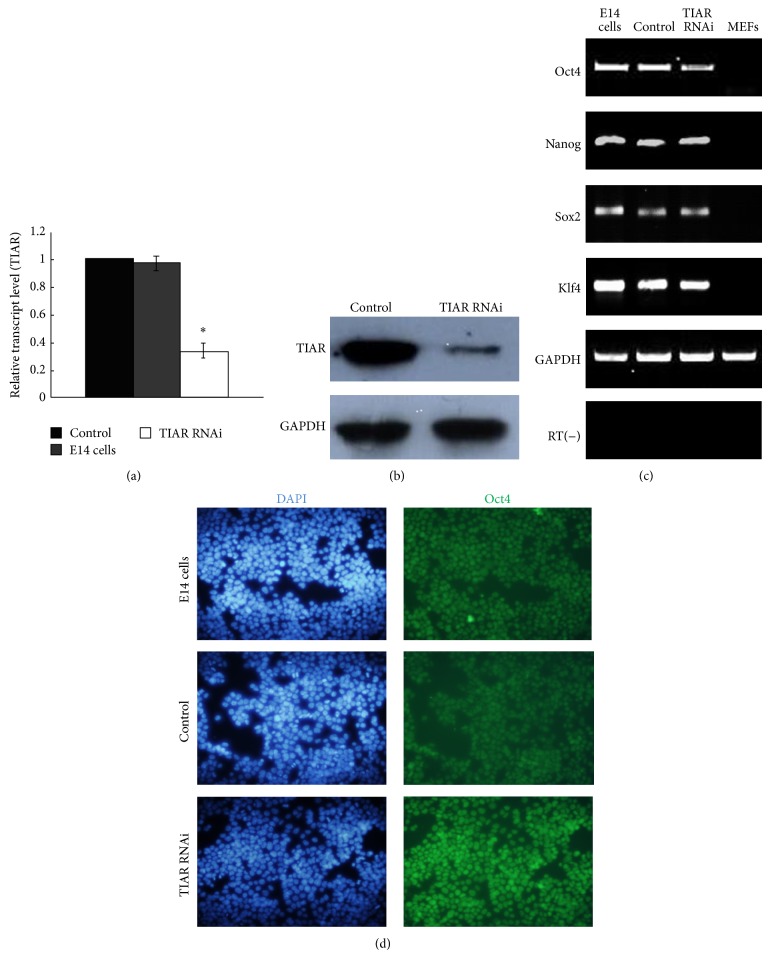
TIAR silencing E14 cells maintained their self-renewal pluripotency. (a) Quantitative RT-PCR analysis of the gene expression of TIAR in TIAR RNAi E14 cells and pSIREN-RetroQ control E14 cells. (b) Western blot analysis of the expression of TIAR in TIAR RNAi E14 cells and pSIREN-RetroQ control E14 cells. (c) RT-PCR analysis of the expression of Oct4, Nanog, Sox2, and Klf4 in TIAR RNAi E14 cells and pSIREN-RetroQ control E14 cells. E14 cells and mouse embryonic fibroblasts (MEFs) were used as a control. (d) Immunofluorescence analysis of the expression of Oct4 in TIAR RNAi E14 cells and pSIREN-RetroQ control E14 cells (200x). ^∗^ *P* < 0.05 by one-way ANOVA. Control: pSIREN-RetroQ control E14 cells; TIAR RNAi: TIAR RNAi E14 cells.

To investigate the function of TIAR in mouse ESCs, we downregulated its expression in E14 cells via RNA interference (RNAi) using siRNA. Transfections were carried out using Lipofectamine 2000 (Invitrogen) and pSIREN-RetroQ-TIAR-shRNA plasmid, which contains a short hairpin RNA (shRNA) sequence that targets mouse TIAR mRNA. In consideration of the fact that TIA-1 shares an 80% amino acid homology with TIAR, sequence alignment of the cDNA of TIAR and TIA-1 was used. Thus sequence homology of TIAR and TIA-1 was avoided and siRNA sequence selected at this position was absolutely targeting TIAR. A shRNA nonsilencing vector was used as a control. We constructed stably transfected TIAR RNAi E14 clones and control E14 clones. Quantitative RT-PCR (qRT-PCR) and Western blot analysis were conducted to evaluate TIAR expression in these two cell lines (Figures 1(a) and 1(b)); TIAR abundance in TIAR RNAi E14 cells was effectively reduced, relative to control cells. Both groups expressed the key molecular markers found in mouse ESCs—Oct4, Nanog, Sox2, and Klf4 (Figures 1(c) and 1(d)) [20, 21]—thus confirming their pluripotent status.

### 3.2. Downregulation of TIAR Protein Levels Restrained the Differentiation of E14 Cells

**Figure 2:**
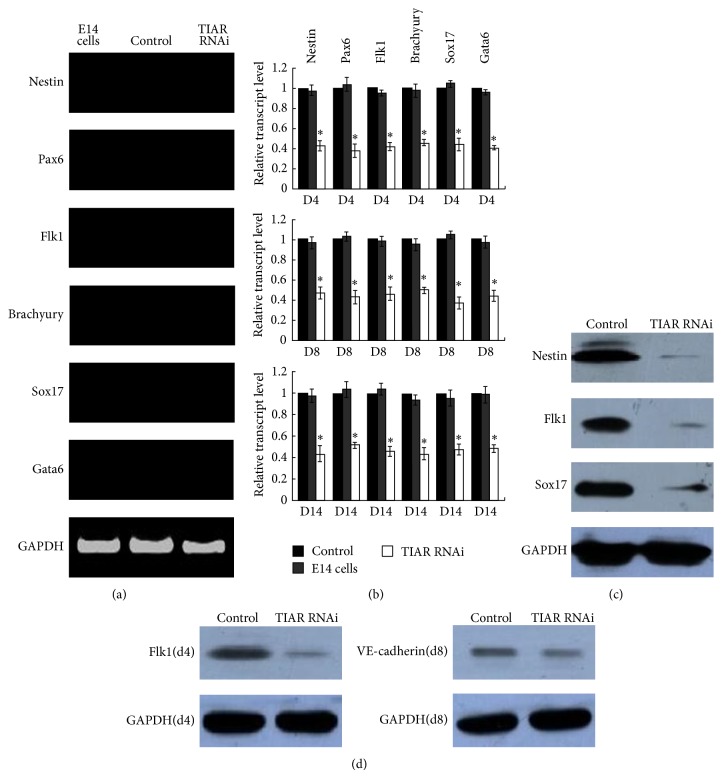
Downregulation of TIAR protein levels restrained the differentiation of E14 cells. (a) RT-PCR analysis of the gene expression of nestin, Pax6, Flk-1, brachyury, Sox17, and Gata6 on day 0 in TIAR RNAi E14 cells and pSIREN-RetroQ control E14 cells. E14 cells were used as control. (b) Quantitative RT-PCR analysis of the gene expression of nestin, Pax6, Flk-1, brachyury, Sox17, and Gata6 on day 4, day 8, and day 14 during EB differentiation in TIAR RNAi E14 cells and pSIREN-RetroQ control E14 cells. Day 4, day 8, and day 14 EB differentiation of E14 cells were used as control. (c) Western blot analysis of the gene expression of nestin, Flk-1, and Sox17 on day 4 during EB differentiation in TIAR RNAi E14 cells and pSIREN-RetroQ control E14 cells. (d) Western blot analysis of the gene expression of Flk-1 (day 4) and VE-cadherin (day 8) in monolayer differentiation of TIAR RNAi E14 cells and pSIREN-RetroQ control E14 cells. ^∗^ *P* < 0.05 by one-way ANOVA. Control: pSIREN-RetroQ control E14 cells; TIAR RNAi: TIAR RNAi E14 cells.

The classical method to induce ESC differentiation is to allow them to grow in suspension and form three-dimensional aggregates known as embryoid bodies (EBs) [22]. However before differentiation of mouse TIAR RNAi E14 cells, the expression of neuroectoderm markers Pax6 and nestin, mesoderm markers brachyury and Flk1, and hypoblast and definitive endoderm markers Sox17 and Gata6 [23] was firstly measured via RT-PCR on undifferentiated day 0. While no expression levels of marker gene were detected (Figure 2(a)), we confirmed that on undifferentiated day 0 no differentiation abilities have been promoted. To determine the effect of TIAR silencing on the differentiation of E14 cells, the expression levels of specific marker gene were all assessed via qRT-PCR during EBs differentiation on days 4 and 8 in both TIAR RNAi E14 cells and control cells. Gene expression levels in TIAR RNAi E14 cells were all significantly reduced relative to control cells (Figure 2(b)) (Table 1). Evaluation of the expression of nestin, Flk1, and Sox17 via Western blot analysis supported our findings (Figure 2(c)).

Moreover, to investigate the effect of TIAR knockdown on relatively longer differentiation time of stably transfected TIAR RNAi E14 cells clones, via puromycin selection every two days, we detected the expression levels of differentiation marker gene during EB differentiation on day 14 via qRT-PCR. The gene expression levels in TIAR RNAi E14 cells were all highly decreased relative to control cells (Figure 2(b)) (Table 1), and we confirmed that EB differentiation was restrained in E14 cells after TIAR knockdown.

To further study alterations in the differentiation of E14 cells as a result of TIAR knockdown, TIAR RNAi E14 cells were induced into endothelial cells (EC) with monolayer differentiation methods. We assessed the expression of Flk-1 which is a marker of lateral plate mesoderm [24] and the earliest marker for endothelial and blood cells [25, 26] in TIAR RNAi E14 and control cells on day 4 using Western blot assay. Expression of Flk-1 in TIAR RNAi E14 cells was dramatically reduced relative to control cells. We next assessed expression levels of VE-cadherin, marker of endothelial cells [27], on day 8 of differentiation and observed a similar decrease in expression in TIAR RNAi E14 cells (Figure 2(d)).

### 3.3. Downregulation of TIAR Protein Levels Stimulates the Expression of c-myc and TIA-1 in E14 Cells

**Figure 3:**
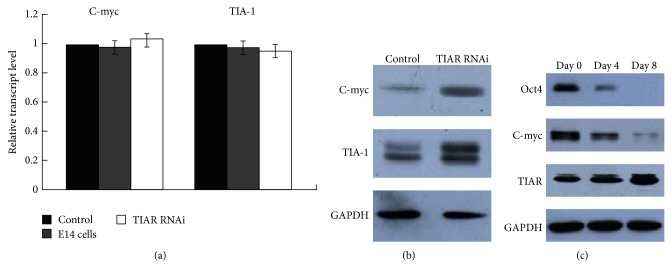
Downregulation of TIAR protein levels stimulates the expression of c-myc and TIA-1 in E14 cells. (a) Quantitative RT-PCR analysis of the gene expression of c-myc and TIA-1 in TIAR RNAi E14 cells and pSIREN-RetroQ control E14 cells. E14 cells were used as a control. (b) Western blot analysis of the gene expression of c-myc and TIA-1 in TIAR RNAi E14 cells and pSIREN-RetroQ control E14 cells. (c) Western blot analysis of the gene expression of Oct4, c-myc, and TIAR in undifferentiated TIAR RNAi E14 cells (day 0) and differentiated cell of TIAR RNAi E14 cells (day 4 and day 8). Control: pSIREN-RetroQ control E14 cells; TIAR RNAi: TIAR RNAi E14 cells.

To investigate the function of TIAR as a translational repressor of c-myc in E14 cells, we examined expression levels of c-myc RNA and protein in TIAR RNAi E14 and control cells. As shown in Figure 3(b), c-myc protein levels were highly increased in TIAR RNAi E14 cells relative to control. However, no measurable changes in c-myc RNA levels were observed in either group (Figure 3(a)). These data support the view that TIAR functions as a translational suppressor of c-myc. As such, we proposed that downregulating TIAR protein levels promoted cell proliferation and pluripotency possibly by stimulating the expression of c-myc.

To examine additional TIAR target mRNAs, we assayed the expression of TIA-1 RNA and protein. As shown in Figure 3(b), expression of TIA-1 protein levels was not reduced and remained upregulated in TIAR RNAi E14 cells. However, similar to that of c-myc, no measurable changes were observed in TIA-1 RNA levels (Figure 3(a)).

To further probe the functions of TIAR and c-myc in TIAR RNAi E14 cells, we measured TIAR and c-myc expression levels in undifferentiated (day 0) and differentiated (days 4 and 8) EBs generated from TIAR RNAi E14 cells via Western blot. c-myc was significantly downregulated during differentiation, whereas the expression of TIAR was increased (Figure 3(c)). Our results suggest that the reduction of c-myc during differentiation was largely due to TIAR as TIAR inhibits the translation of c-myc. Therefore, one can surmise that, in this context, TIAR promotes differentiation at least in part by inhibiting the expression of c-myc at the posttranscriptional level.

### 3.4. Expression of Exogenous RNAi-Resistant TIAR Rescues the Function of TIAR in siRNA-Treated Cells

To confirm the specificity of our RNAi construct, we evaluated whether the observed effects resulting from the knockdown of TIAR could be rescued by exogenous expression of TIAR in siRNA-treated cells. We transfected TIAR RNAi E14 cells with the full-coding sequence of TIAR, in which a short stretch of sequence, complementary to the siRNA oligonucleotide, was mutated to prevent the destruction of exogenous mRNA. TIAR RNAi E14 cells were infected with a pCDNA3.1-Hygro vector containing the RNAi-resistant TIAR transgene. TIAR RNAi E14 cells were infected with an empty pCDNA3.1-Hygro vector as a control.

**Figure 4:**
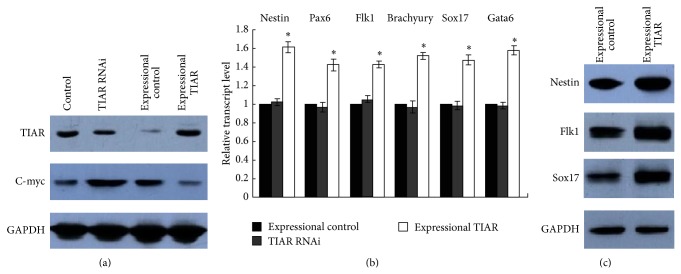
Expression of exogenous RNAi-resistant TIAR rescues the function of TIAR in siRNA-treated cells. (a) Western blot analysis of the expression of TIAR and c-myc in control, TIAR RNAi, expressional control, and expressional TIAR. (b) Quantitative RT-PCR analysis of the gene expression of nestin, Pax6, Flk-1, brachyury, Sox17, and Gata6 on day 4 during EB differentiation in TIAR RNAi, expressional control, and expressional TIAR. (c) Western blot analysis of the gene expression of nestin, Flk-1, and Sox17 on day 4 during EB differentiation in expressional control and expressional TIAR. ^∗^ *P* < 0.05 by one-way ANOVA. Control: E14 cells infected with an empty control pCDNA3.1-Hygro vector; TIAR RNAi: TIAR RNAi E14 cells; expressional control: TIAR RNAi E14 cells infected with an empty control pCDNA3.1-Hygro vector; expressional TIAR: TIAR RNAi E14 cells infected with pCDNA3.1-Hygro vector containing RNAi-resistant TIAR transgene.

As shown in Figure 4(a), we performed Western blot analyses to assess the expression of TIAR and c-myc in TIAR siRNA-treated cells. Whereas the expression of TIAR increased in cells infected with the RNAi-resistant TIAR transgene, the expression of c-myc decreased. However, on the other hand, in TIAR RNAi E14 cells infected with an empty control pCDNA3.1 vector, the expression of c-myc increased. As shown in Figure 4(b) (Table 2) and Figure 4(c), the reduced differentiation observed after knockdown of TIAR was rescued via exogenous expression of the RNAi-resistant TIAR transgene in siRNA-treated cells.

## 4. Discussion

Whereas LIF/STAT3 signaling plays an important role to murine ESC maintenance, previous work [28] has demonstrated that bone morphogenic proteins (BMPs) may work in collaboration with LIF to promote self-renewal. Only recently, Ying et al. [29] found that self-renewal of mouse ESCs does not require activating signals from LIF/STAT3 and BMP/SMAD pathways but rather using a combination of small-chemical molecules inhibits the mitogen-activated protein kinase (MAPK)/extracellular signal-related kinase (ERK1/2) and the glycogen synthase kinase 3 (GSK3). Under these conditions (2i), it was possible to maintain self-renewal in the absence of LIF/STAT3 stimulation.

In this study we utilized RNAi and a subsequent genetic rescue to analyze the function of RNA-binding protein TIAR in mouse E14 ESCs, with respect to self-renewal and differentiation. We observed that knockdown of TIAR expression resulted in sustained pluripotency and self-renewal capabilities but restrained ESC-derived EB differentiation.

Through evaluation of neuroectoderm markers Pax6 and nestin, mesoderm markers brachyury and Flk1, and hypoblast and definitive endoderm markers Gata6 and Sox17 in differentiated EBs (day 4, day 8, and longer time on day 14), we provided evidence that TIAR knockdown restrained the differentiation of E14-derived EBs. We have thus concluded that downregulation of TIAR expression attenuated EB differentiation into the three germ layers.

We assessed the gene expression levels of Flk-1 on day 4 and VE-cadherin on day 8 of monolayer differentiation and confirmed that downregulation of TIAR attenuated the differentiation of E14 cells particularly into endothelial cells.

As expected, due to TIAR-mediated inhibition of c-myc, we found that knockdown of TIAR influenced the capacity for self-renewal in E14 cells. Myc proteins are known to be crucial for stem cell maintenance and related; c-myc is a target of the LIF/STAT3 pathway. Recent studies with c-myc/N-myc double-knockout (dKO) ESCs demonstrate that c-myc and N-myc are required to maintain ESC self-renewal and pluripotency [30]. Loss of c- and N-myc strongly induces differentiation into ectoderm, mesoderm, and endoderm derivatives. In our TIAR RNAi E14 cells group, expression of exogenous RNAi-resistant TIAR, thus the expression of c-myc decreased, the abilities of EB differentiation of E14 cells were highly promoted. However, overexpression of myc inhibits differentiation of a variety of cell types including hematopoietic and neuronal cells [31], and an examination of myc molecular function in ESCs showed that myc sustains pluripotency by repressing gene expression of the primitive endoderm master regulator Gata6 [32]. In our TIAR RNAi E14 cells group, the gene expression of Gata6 was also restrained. Moreover, c-myc acts, in part, through a subset of miRNAs to attenuate differentiation in ESCs [33]. Introduction of c-myc miRNAs into murine ESCs significantly attenuates the expected downregulation of pluripotency markers typically observed upon withdrawal of LIF. In contrast, knockdown of endogenous miRNAs accelerates differentiation.

Based on these findings, we proposed that downregulation of TIAR restrained the differentiation of E14-derived EBs in part due to its downstream interactions with c-myc. Our study identified that RNA-binding protein TIAR inhibited c-myc expression at the translational level in E14 ESCs, and as a result TIAR knockdown E14 cells promoted enhanced self-renewal and pluripotency in addition to attenuated differentiation.

Mazan-Mamczarz et al. [34] revealed that, in human RKO colorectal carcinoma cells, TIA-1 expression levels remained unaltered after TIAR silencing. Pullmann Jr. et al. [19] used human cervical carcinoma HeLa cells to test the hypothesis that the turnover and translation of regulatory RNA-binding proteins (TTR-RBP), including AUF1, HuR, KSRP, NF90, TIA-1, and TIAR, were influenced posttranscriptionally by TTR-RBPs themselves. They found that TIAR knockdown upregulated TIA-1 protein levels and, conversely, TIA-1 knockdown upregulated TIAR protein levels. In agreement with previous research, we found that in mouse E14 ESCs TIAR inhibited TIA-1 expression at the translational level. Furthermore, compared with control cells the expression of TIA-1 protein levels was not reduced and remained upregulated in TIAR RNAi E14 cells. However TIA-1 RNA levels in these two groups were not changed. Our findings suggested that as the sequence homology of TIAR and TIA-1 was avoided, the influence of knockdown of TIAR gene obtained using the siRNA sequence was absolutely due to TIAR silencing, suggesting that observed effect is not off-target.

TIAR has a distinct role in mediating the translational silencing of ARE-bound mRNAs, particularly in cells responding to environmental stressors. Furthermore, TIAR is a multifunctional RNA-binding protein [35, 36] that both participates in pre-mRNA splicing [37] and promotes apoptosis [38]. As TIAR is highly expressed in primordial germ cells (PGCs) and mutant mice lacking TIAR fail to develop spermatogonia or oogonia [17], it is inferred that TIAR contributes to embryo development. However, whereas a role for TIAR has been implicated in many cellular functions, the precise underlying mechanisms have yet to be elucidated. Here, we have demonstrated a role for TIAR, via posttranscriptional regulation of targets ARE-c-myc mRNA, in the control of self-renewal and differentiation of mouse ESCs. The precise mechanism by which TIAR binds to and represses the translation of specific target ARE-containing mRNAs remains unclear. As such, the analysis of possible posttranslational modifications of TIAR that modulate its association with target mRNAs, particularly in mouse ESCs, is likely to be a fruitful area of future pursuit.

Here, using RNAi and subsequent genetic rescue, we have demonstrated an indirect relationship between TIAR and c-myc expression levels and shed light on a downstream pathway leading to the maintenance of ESC self-renewal, pluripotency, and differentiation. Moreover, we confirmed a regulatory relationship existing between RNA-binding proteins TIAR and TIA-1.

## 5. Conclusion

We used RNAi and genetic rescue assays to analyze the functions of RNA-binding protein TIAR in mouse E14 ESCs. We demonstrated that TIAR inhibited c-myc expression at the translational level in mouse E14 cells; accordingly, RNAi-mediated TIAR knockdown promoted self-renewal of cells and maintained a pluripotent state, thereby attenuating differentiation. Moreover, we supplied evidence that TIAR inhibited TIA-1 expression at the translational level, thus providing insight into the functional relationship that exists between the two RNA-binding proteins.

## Supplementary Material

Supplementary Material: RNAi and subsequent rescue expression of the RNA binding protein TIAR in mouse E14 ESCs.

## Figures and Tables

**Table 1:** Gene expression during EB differentiation in control and TIAR RNAi E14 cells.

	E14 cells	Control	TIAR RNAi
Day 4			
Nestin	0.98 ± 0.051	1.0	0.43 ± 0.050^∗^
Pax6	1.04 ± 0.070	1.0	0.38 ± 0.065^∗^
Flk1	0.95 ± 0.031	1.0	0.42 ± 0.045^∗^
Brachyury	0.98 ± 0.066	1.0	0.46 ± 0.035^∗^
Sox17	1.06 ± 0.015	1.0	0.44 ± 0.061^∗^
Gata6	0.96 ± 0.025	1.0	0.41 ± 0.021^∗^
Day 8			
Nestin	0.97 ± 0.059	1.0	0.47 ± 0.060^∗^
Pax6	1.03 ± 0.045	1.0	0.43 ± 0.067^∗^
Flk1	0.99 ± 0.050	1.0	0.46 ± 0.064^∗^
Brachyury	0.95 ± 0.061	1.0	0.50 ± 0.035^∗^
Sox17	1.05 ± 0.040	1.0	0.37 ± 0.055^∗^
Gata6	0.97 ± 0.065	1.0	0.44 ± 0.057^∗^
Day 14			
Nestin	0.98 ± 0.065	1.0	0.44 ± 0.071^∗^
Pax6	1.04 ± 0.076	1.0	0.52 ± 0.030^∗^
Flk1	1.04 ± 0.055	1.0	0.46 ± 0.045^∗^
Brachyury	0.94 ± 0.045	1.0	0.44 ± 0.059^∗^
Sox17	0.96 ± 0.070	1.0	0.48 ± 0.050^∗^
Gata6	0.99 ± 0.076	1.0	0.49 ± 0.036^∗^

^∗^P < 0.05 by one-way ANOVA. Control: pSIREN-RetroQ control E14 cells; TIAR RNAi: TIAR RNAi E14 cells.

**Table 2:** Gene expression on day 4 EB differentiation in rescue TIAR siRNA-treated cells.

	TIAR RNAi	Expressional control	Expressional TIAR
Nestin	1.03 ± 0.032	1.0	1.62 ± 0.057^∗^
Pax6	0.97 ± 0.055	1.0	1.43 ± 0.060^∗^
Flk1	1.06 ± 0.036	1.0	1.43 ± 0.035^∗^
Brachyury	0.97 ± 0.065	1.0	1.53 ± 0.040^∗^
Sox17	0.99 ± 0.047	1.0	1.48 ± 0.051^∗^
Gata6	0.99 ± 0.042	1.0	1.58 ± 0.045^∗^

^∗^ *P* < 0.05 by one-way ANOVA. TIAR RNAi: TIAR RNAi E14 cells; expressional control: TIAR RNAi E14 cells infected with an empty control pCDNA3.1-Hygro vector; expressional TIAR: TIAR RNAi E14 cells infected with pCDNA3.1-Hygro vector containing RNAi-resistant TIAR transgene.

**Table 3:** Primers used for reverse-transcription polymerase chain reaction.

Gene	Sense primer (5′-3′)	Antisense primer (5′-3′)	Annealing temperature (°C)	Size (bp)

Oct4	GCTCAGCCTTAAGAACATGTGTAAGC	GCCTCATACTCTTCTCGTTGGGA	56	327
Nanog	ATGAAGTGCAAGCGGTGGCAGAAA	CCTGGTGGAGTCACAGAGTAGTTC	56	464
Sox2	GGCGGCAACCAGAAGAACAG	GTTGCTCCAGCCGTTCATGTG	56	414
Klf4	CAGTCGCAAGTCCCCTCTCTC	CCTGTCGCACTTCTGGCACTG	56	321
GAPDH	ACTCACGGCAAATTCAACGG	ACGTCAGATCCACGACGGAC	56	586

**Table 4:** Primers used for quantitative reverse-transcription polymerase chain reaction.

Gene	Sense primer (5′-3′)	Antisense primer (5′-3′)
TIAR	TAACAGAGCAACCCGATAGCAGA	TCCACAAAGCAATATGGGTCATT
Nestin	CTGAATGAAGGAGGTATGG	CACAATGGCTCTTGAGTATC
Pax6	CAAACCTGTCTCCTCCTTCACA	GGTGAGGGCGGTGTCTGT
Flk-1	GATCGGTGAGAAAGCCTTGATCT	CTCCATTCTTTACAAGCATACGG
Brachyury	CGAGATGATTGTGACCAAGAACG	CACGAAGTCCAGCAAGAAAGAGTA
Sox17	AGAGCTAAGCAAGATGCTAGGCA	GGTACTTGTAGTTGGGGTGGTCC
Gata6	CTCTACAGCAAGATGAATGGCCTCA	GCTCACCCTCAGCATTTCTACGC
C-myc	GGGACAGTGTTCTCTGCCTCT	TTCTCTTCCTCGTCGCAGAT
TIA-1	AACAGACTACAGAACGGAAGC	GCAAACATCCACCATCGT
RPL19	GACGGAAGGGCAGGCATATG	TGTGGATGTGCTCCATGAGG
